# Supplementary material for: Cell-type-specific Alzheimer’s disease polygenic risk scores are associated with distinct disease processes in Alzheimer’s disease
Source: Nat Commun. 2023 Nov 30;14:7659. doi: 10.1038/s41467-023-43132-2 (PMC10689816; doi:10.1038/s41467-023-43132-2)
Supplement: Supplementary file 1 — Supplementary Information [file 41467_2023_43132_MOESM1_ESM.pdf]

Supplementary materials to:
Cell-type-specific Alzheimer's disease polygenic risk scores are associated with distinct disease processes in Alzheimer's disease

Hyun-Sik Yang[1,2,3,4,*], Ling Teng[1,4], Daniel Kang[2], Vilas Menon[5,6], Tian Ge[3,4,7,8], Hilary K. Finucane[3,4,9], Aaron P. Schultz[2,3], Michael Properzi[2], Hans-Ulrich Klein[5,6], Lori B. Chibnik[2,4,10], Julie A. Schneider[11], David A. Bennett[11], Timothy J. Hohman[12], Richard P. Mayeux[6], Keith A. Johnson[1,2,3,13], Philip L. De Jager[5,6], Reisa A. Sperling[1,2,3]

[1]Center for Alzheimer Research and Treatment, Department of Neurology, Brigham and Women's Hospital, Boston, MA, USA
[2]Department of Neurology, Massachusetts General Hospital, Boston, MA, USA
[3]Harvard Medical School, Boston, MA
[4]Broad Institute of MIT and Harvard, Cambridge, MA
[5]Center for Translational & Computational Neuroimmunology, Department of Neurology, Columbia University Irving Medical Center, New York, NY, USA
[6]Department of Neurology and the Taub Institute for the Study of Alzheimer's Disease and the Aging Brain, Columbia University Irving Medical Center, New York, NY, USA
[7]Psychiatric and Neurodevelopmental Genetics Unit, Center for Genomic Medicine, Massachusetts General Hospital, Boston, MA.
[8]Center for Precision Psychiatry, Department of Psychiatry, Massachusetts General Hospital, Boston, MA, USA
[9]Department of Medicine, Massachusetts General Hospital, Boston, MA, USA
[10]Department of Epidemiology, Harvard T.H. Chan School of Public Health, Boston, MA, USA
[11]Rush Alzheimer's Disease Center, Rush University Medical Center, Chicago, IL, USA
[12]Department of Neurology, Vanderbilt University Medical Center, Nashville, TN, USA
[13]Department of Radiology, Massachusetts General Hospital, Boston, MA, USA

*Corresponding Author: Hyun-Sik Yang, MD
E-mail: hyang18@bwh.harvard.edu

Cell type	N_SNP_ROSMAP (%)	N_SNP_A4 (%)	N_SNP_HRC (%)	N_SNP_PRSet (%)
Ex	78555 (7.6)	81405 (7.6)	445321 (6.8)	20283 (10.7)
In	101104 (9.7)	104006 (9.7)	578298 (8.8)	25334 (13.3)
Ast	82828 (8.0)	85708 (8.0)	458050 (7.0)	21729 (11.4)
Mic	71283 (6.9)	74069 (6.9)	401171 (6.1)	19158 (10.1)
Oli	82719 (8.0)	85342 (8.0)	473174 (7.2)	20452 (10.8)
Opc	108157 (10.4)	111476 (10.4)	609520 (9.3)	26966 (14.2)
All[a]	1039252 (100.0)	1067306 (100.0)	6569519 (100.0)	190005 (100.0)

Supplementary Table 1. Number of SNPs included in each cell-type-specific ADPRS. The number and proportion of the post-LD shrinkage SNPs (i.e., PRS-CS-processed SNPs) included in each cell-type-specific ADPRS are shown for ROSMAP (N_SNP_ROSMAP) and A4 (N_SNP_A4). Each cell-type-specific ADPRS includes SNPs within cell-type-specific genomic regions (1,343 cell-type-specific genes per each cell type ± 30 kb margins). While the exact numbers of N_SNP_ROSMAP and N_SNP_A4 are slightly different (<5% difference due to genotype missingness in each dataset), the proportions of SNPs included in each cell-type-specific ADPRS were highly consistent. For comparison, total HRC-imputed SNP count before LD shrinkage (N_SNP_HRC) and after LD pruning (N_SNP_PRSet, p-value threshold=1) are also shown for the ROSMAP genotype data. Although LD shrinkage using PRS-CS was limited to the HapMap3 SNPs (N_SNP_ROSMAP and N_SNP_A4), it retains more SNPs with posterior effect sizes than the LD pruning approach (N_SNP_PRSet). [a]All autosomal SNPs excluding the *APOE* region.

	Mean (s.d.)	N_nonmissing
AD dementia, n (%)	538 (68.4)	786
Amyloid-β (Aβ) (sqrt)	1.7 (1.1)	1381
Diffuse plaque (DP) (sqrt)	0.71 (0.49)	1452
Neuritic plaque (NP) (sqrt)	0.77 (0.53)	1452
PHFtau (sqrt)	2.3 (1.4)	1451
Neurofibrillary tangle (NFT) (sqrt)	0.70 (0.43)	1452
Cognitive decline	-0.017 (0.094)	1374

Supplementary Table 2. AD endophenotypes tested in ROSMAP. The mean and standard deviation (s.d.) of the AD endophenotypes tested for their associations with cell-type-specific ADPRSs in ROSMAP are shown. For AD dementia (binary trait), we indicated the number of cases and the proportion out of the case (AD dementia) + control (cognitively unimpaired, no AD pathology) subset used for the analyses with AD dementia as the outcome (n=786). Abbreviations: N_nonmissing, number of participants with non-missing data; sqrt, square root-transformed values

Model	OR	95% CI	z-value	p-value	FDR	ΔR^2
**All**	**1.53**	**1.28 to 1.85**	**4.52**	**6.2×10^{-6}**	**3.4×10^{-5}**	**0.028**
Ex	1.04	0.87 to 1.24	0.44	0.66	0.68	NA
In	1.11	0.92 to 1.35	1.12	0.26	0.35	NA
Ast	1.18	0.98 to 1.42	1.74	0.082	0.13	NA
**Mic**	**1.45**	**1.20 to 1.75**	**3.86**	**1.1×10^{-4}**	**3.9×10^{-4}**	**0.020**
Oli	1.26	1.05 to 1.51	2.47	0.014	0.030	NA
Opc	1.05	0.88 to 1.25	0.54	0.59	0.64	NA
APOE ε4	6.81	4.31 to 11.2	7.88	3.3×10^{-15}	NA	0.12
APOE ε2	0.38	0.24 to 0.60	-4.17	3.1×10^{-5}	NA	0.023

Supplementary Table 3. Association between cell-type-specific ADPRS and AD dementia in ROSMAP (case: n=538, control: n=248). OR (odds ratio) of AD dementia per 1 s.d. increase in ADPRS is shown. ADPRS models (logistic regression) were adjusted for *APOE* ε4, *APOE* ε2, age at death, sex, years of education, genotyping platform, and the first three genotype principal components. For the cell-type-specific ADPRS that showed a significant association with the trait (FDR<0.025), variance explained by the PRS was captured by comparing Nagelkerke's R^2 between the models with and without the given PRS term (ΔR^2). For comparison, statistics for *APOE* ε4 and ε2 from the same model as All-ADPRS (with the same covariates) were shown in the bottom two lines of the table. All p-values are two-sided. To account for multiple comparisons, false discovery rate (FDR) correction was applied across all main tests in ROSMAP (**Supplementary Tables 3-9**), and statistically significant results (FDR<0.025) were indicated in bold. (Also see **Fig. 2**).

Model	Beta	95% CI	t-value	p-value	FDR	ΔR^2
**All**	**0.081**	**0.027 to 0.14**	**2.92**	**3.6×10^{-3}**	**9.3×10^{-3}**	**4.7×10^{-3}**
Ex	0.020	-0.034 to 0.075	0.73	0.46	0.53	NA
In	-0.028	-0.082 to 0.027	-0.98	0.33	0.41	NA
**Ast**	**0.093**	**0.039 to 0.15**	**3.37**	**7.8×10^{-4}**	**2.6×10^{-3}**	**6.5×10^{-3}**
Mic	0.057	2.4×10^{-3} to 0.11	2.05	0.041	0.074	NA
Oli	0.055	1.1×10^{-3} to 0.11	2.00	0.045	0.079	NA
Opc	0.014	-0.041 to 0.068	0.49	0.62	0.66	NA
APOE ε4	0.65	0.53 to 0.76	11.1	2.1×10^{-27}	NA	0.077
APOE ε2	-0.35	-0.50 to -0.21	-4.73	2.5×10^{-6}	NA	0.014

Supplementary Table 4. Association between cell-type-specific ADPRS and Aβ in ROSMAP (n=1,381). Beta (effect size) corresponds to units changed in Aβ per 1 s.d. increase in ADPRS. ADPRS models (linear regression) were adjusted for *APOE* ε4, *APOE* ε2, age at death, sex, genotyping platform, and the first three genotype principal components. For the cell-type-specific ADPRS that showed a significant association with the trait (FDR<0.025), variance explained by the PRS was captured by comparing adjusted R^2 between the linear models with and without the given PRS term (ΔR^2). For comparison, the statistics for *APOE* ε4 and ε2 from the same model as All-ADPRS (with the same covariates) were shown in the bottom two lines of the table. All p-values are two-sided. To account for multiple comparisons, false discovery rate (FDR) correction was applied across all main tests in ROSMAP (**Supplementary Tables 3-9**), and statistically significant results (FDR<0.025) were indicated in bold. (Also see **Fig. 2**).

Model	Beta	95% CI	t-value	p-value	FDR	ΔR^2
All	0.013	-0.011 to 0.037	1.04	0.30	0.39	NA
Ex	-6.8×10^{-3}	-0.031 to 0.018	-0.55	0.58	0.64	NA
In	-0.011	-0.036 to 0.013	-0.92	0.36	0.43	NA
**Ast**	**0.034**	**9.8×10^{-3} to 0.058**	**2.75**	**6.0×10^{-3}**	**0.014**	**4.1×10^{-3}**
Mic	0.016	-8.2×10^{-3} to 0.040	1.30	0.19	0.26	NA
Oli	0.026	1.9×10^{-3} to 0.050	2.12	0.034	0.070	NA
Opc	0.012	-1.3×10^{-3} to 0.036	0.93	0.35	0.43	NA
APOE ε4	0.26	0.21 to 0.31	9.93	1.6×10^{-22}	NA	0.061
APOE ε2	-0.14	-0.21 to -0.076	-4.28	2.0×10^{-5}	NA	0.011

Supplementary Table 5. Association between cell-type-specific ADPRS and diffuse plaque burden in ROSMAP (n=1,452). Beta (effect size) corresponds to units changed in diffuse plaque burden per 1 s.d. increase in ADPRS. ADPRS models (linear regression) were adjusted for *APOE* ε4, *APOE* ε2, age at death, sex, genotyping platform, and the first three genotype principal components. For the cell-type-specific ADPRS that showed a significant association with the trait (FDR<0.025), variance explained by the PRS was captured by comparing adjusted R^2 between the linear models with and without the given PRS term (ΔR^2). For comparison, the statistics for *APOE* ε4 and ε2 from the same model as All-ADPRS (with the same covariates) were shown in the bottom two lines of the table. All p-values are two-sided. To account for multiple comparisons, false discovery rate (FDR) correction was applied across all main tests in ROSMAP (**Supplementary Tables 3-9**), and statistically significant results (FDR<0.025) were indicated in bold. (Also see **Fig. 2**).

Model	Beta	95% CI	t-value	p-value	FDR	ΔR^2
**All**	**0.059**	**0.036 to 0.085**	**4.53**	**6.3×10^{-6}**	**3.4×10^{-5}**	**0.012**
Ex	0.020	-6.1×10^{-3} to 0.045	1.50	0.13	0.19	NA
In	1.2×10^{-4}	-0.026 to 0.026	8.8×10^{-3}	0.99	0.99	NA
**Ast**	**0.051**	**0.026 to 0.077**	**3.94**	**8.4×10^{-5}**	**3.2×10^{-4}**	**8.6×10^{-3}**
**Mic**	**0.055**	**0.029 to 0.080**	**4.20**	**2.8×10^{-5}**	**1.3×10^{-4}**	**9.9×10^{-3}**
**Oli**	**0.056**	**0.031 to 0.082**	**4.33**	**1.6×10^{-5}**	**7.7×10^{-5}**	**0.011**
Opc	0.012	-0.014 to 0.038	0.90	0.37	0.43	NA
APOE ε4	0.32	0.27 to 0.38	11.8	1.2×10^{-30}	NA	0.081
APOE ε2	-0.19	-0.25 to -0.12	-5.39	8.4×10^{-8}	NA	0.017

Supplementary Table 6. Association between cell-type-specific ADPRS and neuritic plaque burden in ROSMAP (n=1,452). Beta (effect size) corresponds to units changed in neuritic plaque burden per 1 s.d. increase in ADPRS. ADPRS models (linear regression) were adjusted for *APOE* ε4, *APOE* ε2, age at death, sex, genotyping platform, and the first three genotype principal components. For the cell-type-specific ADPRS that showed a significant association with the trait (FDR<0.025), variance explained by the PRS was captured by comparing adjusted R^2 between the linear models with and without the given PRS term (ΔR^2). For comparison, the statistics for *APOE* ε4 and ε2 from the same model as All-ADPRS (with the same covariates) were shown in the bottom two lines of the table. All p-values are two-sided. To account for multiple comparisons, false discovery rate (FDR) correction was applied across all main tests in ROSMAP (**Supplementary Tables 3-9**), and statistically significant results (FDR<0.025) were indicated in bold. (Also see **Fig. 2**).

Model	Beta	95% CI	t-value	p-value	FDR	ΔR^2
**All**	**0.24**	**0.17 to 0.31**	**6.72**	**2.6×10^{-11}**	**1.3×10^{-9}**	**0.026**
**Ex**	**0.10**	**0.032 to 0.17**	**2.84**	**4.5×10^{-3}**	**0.011**	**4.3×10^{-3}**
In	0.063	-7.3×10^{-3} to 0.13	1.76	0.079	0.13	NA
**Ast**	**0.12**	**0.047 to 0.19**	**3.29**	**1.0×10^{-3}**	**3.0×10^{-3}**	**5.9×10^{-3}**
**Mic**	**0.21**	**0.15 to 0.28**	**6.09**	**1.4×10^{-9}**	**2.3×10^{-8}**	**0.021**
**Oli**	**0.18**	**0.11 to 0.25**	**5.17**	**2.7×10^{-7}**	**2.2×10^{-6}**	**0.015**
Opc	0.087	0.017 to 0.16	2.45	0.014	0.031	NA
APOE ε4	0.77	0.62 to 0.91	10.3	3.9×10^{-24}	NA	0.062
APOE ε2	-0.31	-0.50 to -0.13	-3.35	8.3×10^{-4}	NA	6.0×10^{-3}

Supplementary Table 7. Association between cell-type-specific ADPRS and tau in ROSMAP (n=1,451). Beta (effect size) corresponds to units changed in tau per 1 s.d. increase in ADPRS. ADPRS models (linear regression) were adjusted for *APOE* ε4, *APOE* ε2, age at death, sex, genotyping platform, and the first three genotype principal components. For the cell-type-specific ADPRS that showed a significant association with the trait (FDR<0.025), variance explained by the PRS was captured by comparing adjusted R^2 between the linear models with and without the given PRS term (ΔR^2). For comparison, the statistics for *APOE* ε4 and ε2 from the same model as All-ADPRS (with the same covariates) were shown in the bottom two lines of the table. All p-values are two-sided. To account for multiple comparisons, false discovery rate (FDR) correction was applied across all main tests in ROSMAP (**Supplementary Tables 3-9**), and statistically significant results (FDR<0.025) were indicated in bold. (Also see **Fig. 2**).

Model	Beta	95% CI	t-value	p-value	FDR	ΔR^2
**All**	**0.068**	**0.048 to 0.089**	**6.52**	**9.4×10^{-11}**	**2.3×10^{-9}**	**0.024**
Ex	0.017	-3.6×10^{-3} to 0.038	1.62	0.10	0.16	NA
In	0.015	-5.7×10^{-3} to 0.036	1.42	0.16	0.22	NA
**Ast**	**0.035**	**0.014 to 0.056**	**3.33**	**8.8×10^{-4}**	**2.7×10^{-3}**	**6.1×10^{-3}**
**Mic**	**0.055**	**0.035 to 0.076**	**5.30**	**1.4×10^{-7}**	**1.3×10^{-6}**	**0.016**
**Oli**	**0.049**	**0.029 to 0.070**	**4.70**	**2.8×10^{-6}**	**2.0×10^{-5}**	**0.013**
Opc	0.022	1.1×10^{-3} to 0.043	2.06	0.039	0.074	NA
APOE ε4	0.23	0.19 to 0.28	10.5	5.2×10^{-25}	NA	0.064
APOE ε2	-0.12	-0.17 to -0.066	-4.34	1.5×10^{-5}	NA	0.010

Supplementary Table 8. Association between cell-type-specific ADPRS and neurofibrillary tangle (NFT) burden in ROSMAP (n=1,452). Beta (effect size) corresponds to units changed in neuritic plaque burden per 1 s.d. increase in ADPRS. ADPRS models (linear regression) were adjusted for *APOE* ε4, *APOE* ε2, age at death, sex, genotyping platform, and the first three genotype principal components. For the cell-type-specific ADPRS that showed a significant association with the trait (FDR<0.025), variance explained by the PRS was captured by comparing adjusted R^2 between the linear models with and without the given PRS term (ΔR^2). For comparison, the statistics for *APOE* ε4 and ε2 from the same model as All-ADPRS (with the same covariates) were shown in the bottom two lines of the table. All p-values are two-sided. To account for multiple comparisons, false discovery rate (FDR) correction was applied across all main tests in ROSMAP (**Supplementary Tables 3-9**), and statistically significant results (FDR<0.025) were indicated in bold. (Also see **Fig. 2**).

Model	Beta	95% CI	t-value	p-value	FDR	ΔR^2
**All**	**-0.013**	**-0.018 to -8.6×10^{-3}**	**-5.50**	**4.5×10^{-8}**	**5.5×10^{-7}**	**0.019**
Ex	-4.2×10^{-3}	-9.0×10^{-3} to 5.6×10^{-4}	-1.73	0.084	0.13	NA
In	1.5×10^{-4}	-4.7×10^{-3} to 5.0×10^{-3}	0.061	0.95	0.97	NA
Ast	-5.1×10^{-3}	-9.9×10^{-3} to -2.7×10^{-4}	-2.07	0.038	0.074	NA
**Mic**	**-9.8×10^{-3}**	**-0.015 to -5.1×10^{-3}**	**-4.05**	**5.5×10^{-5}**	**2.3×10^{-4}**	**0.010**
**Oli**	**-7.2×10^{-3}**	**-0.012 to -2.5×10^{-3}**	**-2.95**	**3.2×10^{-3}**	**8.7×10^{-3}**	**5.2×10^{-3}**
Opc	-4.1×10^{-3}	-8.9×10^{-3} to 7.3×10^{-4}	-1.67	0.096	0.15	NA
APOE ε4	-0.053	-0.063 to -0.0043	-10.3	3.9×10^{-24}	NA	0.070
APOE ε2	0.017	4.3×10^{-3} to 0.030	2.63	8.7×10^{-3}	NA	3.9×10^{-3}

Supplementary Table 9. Association between cell-type-specific ADPRS and cognitive decline (CogDec) in ROSMAP (n=1,374). Beta (effect size) corresponds to units changed in CogDec per 1 s.d. increase in ADPRS. ADPRS models (linear regression) were adjusted for *APOE* ε4, *APOE* ε2, genotyping platform, and the first three genotype principal components. For the cell-type-specific ADPRS that showed a significant association with the trait (FDR<0.025), variance explained by the PRS was captured by comparing adjusted R^2 between the linear models with and without the given PRS term (ΔR^2). For comparison, the statistics for *APOE* ε4 and ε2 from the same model as All-ADPRS (with the same covariates) were shown in the bottom two lines of the table. All p-values are two-sided. To account for multiple comparisons, false discovery rate (FDR) correction was applied across all main tests in ROSMAP (**Supplementary Tables 3-9**), and statistically significant results (FDR<0.025) were indicated in bold. (Also see **Fig. 2**).

Model	Beta	95% CI	t-value	p-value
Ex (adjusted for Mic)	0.086	0.017 to 0.16	2.43	0.015
Ast (adjusted for Mic)	0.096	0.027 to 0.17	2.72	6.7×10^{-3}
Oli (adjusted for Mic)	0.091	7.4×10^{-3} to 0.17	2.14	0.033

Supplementary Table 10. Association between cell-type-specific ADPRS and tau in ROSMAP (n=1,451), adjusting for Mic-ADPRS. Beta (effect size) corresponds to units changed in tau per 1 s.d. increase in ADPRS. ADPRS models (linear regression) were adjusted for Mic-ADPRS, *APOE* ε4, *APOE* ε2, age at death, sex, genotyping platform, and the first three genotype principal components. All p-values are two-sided and not adjusted for multiple comparisons.

Model	Beta	95% CI	t-value	p-value
Ex (excluding Mic)	0.10	0.034 to 0.17	2.92	3.6×10^{-3}
Ast (excluding Mic)	0.092	0.022 to 0.16	2.58	9.9×10^{-3}
Oli (excluding Mic)	0.16	0.086 to 0.22	4.37	1.3×10^{-5}

Supplementary Table 11. Association between cell-type-specific ADPRS and tau in ROSMAP (n=1,451), excluding genes overlapping with Mic-ADPRS. Beta (effect size) corresponds to units changed in tau per 1 s.d. increase in ADPRS. Ex-, Ast-, and Oli- ADPRS were calculated after excluding genes overlapping with Mic-ADPRS. ADPRS models (linear regression) were adjusted for *APOE* ε4, *APOE* ε2, age at death, sex, genotyping platform, and the first three genotype principal components. All p-values are two-sided and not adjusted for multiple comparisons.

	ROSMAP (n=201)
Mean Age at Death, years (SD)	89.7 (5.5)
Female (%)	126 (63)
Mean Education, years (SD)	14.6 (2.6)
APOE ε4 carrier (%)	40 (20)
Elevated Aβ (%)	127 (63)
Pathological diagnosis of AD	122 (61)
Median MMSE (IQR)	25 (8.8)
All-cause dementia (%)	76 (38)
AD dementia (%)	62 (31)
Proportion of Activated Microglia (PAM)	0.084 (0.057)

Supplementary Table 12. Study Participant Characteristics (MAP study microglial morphology subset).

Phenotype	Cell Type (Genomic Margin)	Beta or OR	95% CI	t-value	p-value
AD dem	Mic (10 kb)	1.53	1.27 to 1.85	4.37	$1.2{\times}10^{-5}$
AD dem	Mic (100 kb)	1.57	1.30 to 1.91	4.67	$3.0{\times}10^{-6}$
Aβ	Ast (10 kb)	0.080	0.026 to 0.13	2.92	$3.5{\times}10^{-3}$
Aβ	Ast (100 kb)	0.093	0.039 to 0.15	3.37	$7.8{\times}10^{-4}$
DP	Ast (10 kb)	0.032	$7.6{\times}10^{-3}$ to 0.056	2.58	0.010
DP	Ast (100 kb)	0.040	0.016 to 0.064	3.25	$1.2{\times}10^{-3}$
NP	Ast (10 kb)	0.042	0.017 to 0.068	3.25	$1.2{\times}10^{-3}$
NP	Ast (100 kb)	0.056	0.031 to 0.082	4.33	$1.6{\times}10^{-5}$
NP	Mic (10 kb)	0.051	0.025 to 0.076	3.91	$9.8{\times}10^{-5}$
NP	Mic (100 kb)	0.072	0.046 to 0.097	5.56	$3.2{\times}10^{-8}$
NP	Oli (10 kb)	0.060	0.035 to 0.086	4.64	$3.7{\times}10^{-6}$
NP	Oli (100 kb)	0.059	0.034 to 0.085	4.58	$5.1{\times}10^{-6}$
Tau	Ex (10 kb)	0.066	$-3.8{\times}10^{-3}$ to 0.14	1.85	0.064
Tau	Ex (100 kb)	0.15	0.082 to 0.22	4.28	$2.0{\times}10^{-5}$
Tau	Ast (10 kb)	0.11	0.043 to 0.18	3.16	$1.6{\times}10^{-3}$
Tau	Ast (100 kb)	0.12	0.047 to 0.19	3.29	$1.0{\times}10^{-3}$
Tau	Mic (10 kb)	0.21	0.14 to 0.27	5.85	$6.0{\times}10^{-9}$
Tau	Mic (100 kb)	0.24	0.17 to 0.31	6.92	$6.8{\times}10^{-12}$
Tau	Oli (10 kb)	0.18	0.11 to 0.25	5.01	$6.0{\times}10^{-7}$
Tau	Oli (100 kb)	0.19	0.12 to 0.26	5.48	$5.2{\times}10^{-8}$
NFT	Ast (10 kb)	0.032	0.011 to 0.052	3.02	$2.5{\times}10^{-3}$
NFT	Ast (100 kb)	0.034	0.014 to 0.055	3.28	$1.1{\times}10^{-3}$
NFT	Mic (10 kb)	0.050	0.029 to 0.070	4.78	$2.0{\times}10^{-6}$
NFT	Mic (100 kb)	0.066	0.046 to 0.087	6.39	$2.1{\times}10^{-10}$
NFT	Oli (10 kb)	0.048	0.027 to 0.068	4.54	$6.1{\times}10^{-6}$
NFT	Oli (100 kb)	0.051	0.031 to 0.072	4.97	$7.5{\times}10^{-7}$
CogDec	Mic (10 kb)	-0.011	-0.016 to $-6.7{\times}10^{-3}$	-4.72	$2.6{\times}10^{-6}$
CogDec	Mic (100 kb)	-0.013	-0.017 to $-7.8{\times}10^{-3}$	-5.19	$2.4{\times}10^{-7}$
CogDec	Oli (10 kb)	$-7.6{\times}10^{-3}$	-0.012 to $-2.8{\times}10^{-3}$	-3.11	$1.9{\times}10^{-3}$
CogDec	Oli (100 kb)	$-8.4{\times}10^{-3}$	-0.013 to $-3.6{\times}10^{-3}$	-3.45	$5.8{\times}10^{-4}$

Supplementary Table 13. Association between cell-type-specific ADPRS using different genomic margins and AD endophenotypes in ROSMAP. For the significant findings from the ROSMAP main analyses (FDR<0.025 in **Fig. 2**), we performed sensitivity analyses using cell-type-specific ADPRS with different genomic margins (genes ± 10kb or ± 100kb). All models (logistic regression for AD dem outcome, linear regression for others) were adjusted for *APOE* ε4, *APOE* ε2, age, sex, genotyping platform, years of education (only for AD dem and CogDec), and the first three genotype principal components. All p-values are two-sided and not adjusted for multiple comparisons. Abbreviations: AD dem, AD with dementia; CogDec, cognitive decline; OR, odds ratio.

Phenotype	Cell Type	Beta or OR	95% CI	t-value	p-value
AD dem	Mic	1.46	1.21 to 1.77	3.92	8.9×10^{-5}
Aβ	Ast	0.095	0.040 to 0.15	3.42	6.4×10^{-4}
DP	Ast	0.035	0.011 to 0.059	2.83	4.7×10^{-3}
NP	Ast	0.053	0.027 to 0.078	4.03	5.8×10^{-5}
NP	Mic	0.055	0.029 to 0.080	4.22	2.6×10^{-5}
NP	Oli	0.057	0.032 to 0.083	4.39	1.2×10^{-5}
Tau	Ex	0.098	0.028 to 0.17	2.74	6.2×10^{-3}
Tau	Ast	0.12	0.048 to 0.19	3.32	9.4×10^{-4}
Tau	Mic	0.21	0.14 to 0.28	6.06	1.8×10^{-9}
Tau	Oli	0.18	0.11 to 0.25	5.17	2.6×10^{-7}
NFT	Ast	0.035	0.015 to 0.056	3.35	8.3×10^{-4}
NFT	Mic	0.055	0.034 to 0.075	5.25	1.7×10^{-7}
NFT	Oli	0.049	0.029 to 0.070	4.69	3.0×10^{-6}
CogDec	Mic	-9.8×10^{-3}	-0.015 to -5.1×10^{-3}	-4.05	5.5×10^{-5}
CogDec	Oli	-7.0×10^{-3}	-0.012 to -2.2×10^{-3}	-2.85	4.5×10^{-3}

Supplementary Table 14. Association between cell-type-specific ADPRS and AD endophenotypes in ROSMAP, using 10 genotype PCs. For the significant findings from the ROSMAP main analyses (FDR<0.025 in **Fig. 2**), we performed sensitivity analyses adjusting for 10 genotype PCs (instead of 3 PCs adjusted in the main analyses). All models (logistic regression for AD dem outcome, linear regression for others) were adjusted for *APOE* ε4, *APOE* ε2, age, sex, genotyping platform, years of education (only for AD dem and CogDec), and the first three genotype principal components. All p-values are two-sided and not adjusted for multiple comparisons. Abbreviations: AD dem, AD with dementia; CogDec, cognitive decline; OR, odds ratio; PCs, principal components.

Phenotype	Cell Type	OR/Beta	95% CI	t-value	p-value	ΔR^2 (PRSet)	ΔR^2 (PRSCS)
AD dem	Mic	1.16	0.97 to 1.39	1.57	0.12	NA	0.020
Aβ	Ast	0.032	-0.023 to 0.086	1.14	0.25	NA	6.5×10^{-3}
DP	Ast	5.8×10^{-3}	-0.019 to 0.030	0.47	0.64	NA	4.1×10^{-3}
NP	Ast	0.012	-0.014 to 0.038	0.91	0.37	NA	8.6×10^{-3}
NP	Mic	0.039	0.013 to 0.064	2.95	3.3×10^{-3}	4.6×10^{-3}	9.9×10^{-3}
NP	Oli	0.041	0.015 to 0.066	3.12	1.8×10^{-3}	5.2×10^{-3}	0.011
Tau	Ex	0.14	0.068 to 0.21	3.87	1.2×10^{-4}	8.4×10^{-3}	4.3×10^{-3}
Tau	Ast	0.019	-0.051 to 0.088	0.52	0.60	NA	5.9×10^{-3}
Tau	Mic	0.15	0.084 to 0.22	4.32	1.6×10^{-5}	0.011	0.021
Tau	Oli	0.12	0.049 to 0.19	3.34	8.6×10^{-4}	6.1×10^{-3}	0.015
NFT	Ast	7.8×10^{-3}	-0.013 to 0.028	0.74	0.46	NA	6.1×10^{-3}
NFT	Mic	0.036	0.015 to 0.056	3.40	6.9×10^{-4}	6.4×10^{-3}	0.016
NFT	Oli	0.029	8.7×10^{-3} to 0.050	2.79	5.3×10^{-3}	4.1×10^{-3}	0.013
CogDec	Mic	-6.6×10^{-3}	-0.011 to -1.8×10^{-3}	-2.72	6.7×10^{-3}	4.3×10^{-3}	0.010
CogDec	Oli	-4.6×10^{-3}	-9.4×10^{-3} to 1.9×10^{-4}	-1.88	0.060	NA	5.2×10^{-3}

Supplementary Table 15. Association between PRSet-derived ADPRS and AD endophenotypes in ROSMAP. For the significant findings from the ROSMAP main analyses (FDR<0.025 in **Fig. 2**), we benchmarked our approach against PRSet. For the PRSet-derived ADPRS that showed a nominal association with the trait (uncorrected p<0.05), variance explained by the PRS was calculated by comparing adjusted R^2 between the linear models with and without the given PRS term (ΔR^2). ΔR^2 from the main results (using PRS-CS) were shown for comparison. All models (logistic regression for AD dem outcome, linear regression for others) were adjusted for *APOE* ε4, *APOE* ε2, age, sex, genotyping platform, years of education (only for AD dem and CogDec), and the first three genotype principal components. All p-values are two-sided and not adjusted for multiple comparisons.

Model	Effect type	Effect (95% bootstrap CI)	p-value
Ast → DP →NP	ACME	0.023 (6.9×10^{-3} to 0.04)	5.4×10^{-3}
	ADE	0.028 (8.8×10^{-3} to 0.05)	4.0×10^{-3}
	Total effect	0.051 (0.026 to 0.08)	2.0×10^{-4}
	Mediated proportion	0.46 (0.18 to 0.74)	5.6×10^{-3}
Ast → NP → NFT	ACME	0.023 (0.012 to 0.03)	0
	ADE	0.012 (-4.3×10^{-3} to 0.03)	0.15
	Total effect	0.035 (0.015 to 0.05)	2.0×10^{-4}
	Mediated proportion	0.65 (0.38 to 1.24)	2.0×10^{-4}
Mic → NP → NFT	ACME	0.024 (0.013 to 0.03)	0
	ADE	0.031 (0.014 to 0.05)	0
	Total effect	0.054 (0.034 to 0.07)	0
	Mediated proportion	0.44 (0.27 to 0.66)	0
Mic → NFT → CogDec	ACME	-1.8×10^{-3} (-3.0×10^{-3} to 0)	5.8×10^{-3}
	ADE	-5.5×10^{-3} (-9.7×10^{-3} to 0)	6.0×10^{-3}
	Total effect	-7.3×10^{-3} (-0.012 to 0)	2.0×10^{-4}
	Mediated proportion	0.24 (0.080 to 0.56)	2.0×10^{-4}

Supplementary Table 16. Causal mediation analysis (ROSMAP). Mediation models based on linear regression were run using non-parametric bootstrapping over 10,000 simulations, and 95% bootstrap confidence intervals and empiric two-sided p-values are shown. Also see **Fig. 3**. First three models were adjusted for *APOE* ε4, ε2, age at death, sex, education, genotyping batch, and first three genotype principal components (PC1-3). The Mic → NFT → CogDec model was adjusted for neuritic plaque (NP) burden, *APOE* ε4, ε2, genotyping batch, and PC1-3. The slope of cognitive decline (CogDec) was already adjusted for age, sex, and education. Abbreviations: ACME, average causal mediated effects. ADE, average direct effects. CogDec, cognitive decline. DP, diffuse plaque. NFT, neurofibrillary tangle. NP, neuritic plaque.

	Mean (s.d.)	N_nonmissing
Aβ (SUVR)	1.1 (0.19)	2,921
Tau (SUVR)	1.2 (0.11)	302
HV (mm^3)	3.7×10^3 (4.2×10^2)	1,266
PACC (unit)	0.20 (2.5)	2,918

Supplementary Table 17. AD endophenotypes tested in A4. The mean and standard deviation (s.d.) of the AD endophenotypes tested for their associations with cell-type-specific ADPRSs in A4 are shown. Abbreviations: N_nonmissing, number of participants with non-missing data. Abbreviations: HV, hippocampal volume; PACC, Preclinical Alzheimer Cognitive Composite; SUVR, standardized uptake value ratio.

Model	Beta	95% CI	t-value	p-value	FDR	ΔR^2
**All**	**0.019**	**0.012 to 0.025**	**5.73**	**1.1×10^{-8}**	**3.2×10^{-7}**	**8.9×10^{-3}**
**Ex**	**8.6×10^{-3}**	**2.2×10^{-3} to 0.015**	**2.62**	**8.9×10^{-3}**	**0.025**	**1.6×10^{-3}**
In	-6.2×10^{-4}	-7.0×10^{-3} to 5.8×10^{-3}	-0.19	0.85	0.90	NA
**Ast**	**9.6×10^{-3}**	**3.1×10^{-3} to 0.016**	**2.92**	**3.5×10^{-3}**	**0.012**	**2.1×10^{-3}**
**Mic**	**0.017**	**0.011 to 0.024**	**5.35**	**9.3×10^{-8}**	**1.3×10^{-6}**	**7.7×10^{-3}**
**Oli**	**9.9×10^{-3}**	**3.5×10^{-3} to 0.016**	**3.03**	**2.5×10^{-3}**	**0.010**	**2.3×10^{-3}**
Opc	7.8×10^{-3}	1.4×10^{-3} to 0.014	2.37	0.018	0.041	NA
APOE ε4	0.14	0.13 to 0.15	22.9	8.1×10^{-107}	NA	0.15
APOE ε2	-0.028	-0.046 to -9.7×10^{-3}	-2.99	2.8×10^{-3}	NA	2.2×10^{-3}

Supplementary Table 18. Association between cell-type-specific ADPRS and Aβ in A4 (n=2,921). Beta (effect size) corresponds to units changed in florbetapir PET SUVR (cortical composite) per 1 s.d. increase in ADPRS. ADPRS models (linear regression) were adjusted for *APOE* ε4, *APOE* ε2, age, sex, and the first three genotype principal components. For the cell-type-specific ADPRS that showed a significant association with the trait (FDR<0.025), variance explained by the PRS was captured by comparing adjusted R^2 between the linear models with and without the given PRS term (ΔR^2). For comparison, the statistics for *APOE* ε4 and ε2 from the same model as All-ADPRS (with the same covariates) were shown in the bottom two lines of the table. All p-values are two-sided. To account for multiple comparisons, false discovery rate (FDR) correction was applied across all main tests in A4, and statistically significant results (FDR<0.025) were indicated in bold. (Also see **Fig. 4**).

Model	Beta	95% CI	t-value	p-value
Ex (adjusted for Mic)	6.8×10^{-3}	3.6×10^{-4} to 0.013	2.07	0.038
Ast (adjusted for Mic)	8.0×10^{-3}	1.6×10^{-3} to 0.014	2.43	0.015
Oli (adjusted for Mic)	1.3×10^{-3}	-6.1×10^{-3} to 8.7×10^{-3}	0.35	0.73

Supplementary Table 19. Association between cell-type-specific ADPRS and Aβ in A4 (n=2,921), adjusting for Mic-ADPRS. Beta (effect size) corresponds to units changed in florbetapir PET SUVR (cortical composite) per 1 s.d. increase in ADPRS. ADPRS models were adjusted for Mic-ADPRS, *APOE* ε4, *APOE* ε2, age, sex, and the first three genotype principal components. All p-values are two-sided and not adjusted for multiple comparisons.

Model	Beta	95% CI	t-value	p-value
Ex (excluding Mic)	8.5×10^{-3}	2.0×10^{-3} to 0.015	2.58	9.9×10^{-3}
Ast (excluding Mic)	7.9×10^{-3}	1.5×10^{-3} to 0.014	2.42	0.016
Oli (excluding Mic)	6.3×10^{-3}	-1.1×10^{-4} to 0.013	1.93	0.054

Supplementary Table 20. Association between cell-type-specific ADPRS and Aβ in A4 (n=2,921; excluding genes overlapping with Mic-ADPRS). Beta (effect size) corresponds to units changed in Aβ per 1 s.d. increase in ADPRS. Ex-, Ast-, and Oli- ADPRS were calculated after excluding genes overlapping with Mic-ADPRS. ADPRS models were adjusted for *APOE* ε4, *APOE* ε2, age at death, sex, and the first three genotype principal components. All p-values are two-sided and not adjusted for multiple comparisons.

	A4/LEARN Tau subset (n=302)
Mean Age, years (SD)	71.7 (4.7)
Female (%)	183 (61)
Mean Education, years (SD)	16.3 (2.7)
APOE ε4 carrier (%)	164 (54)
Mean Florbetapir, cortical SUVR (SD)	1.29 (0.20)
Mean Flortaucipir, inferior temporal SUVR (SD)	1.53 (0.28)
Elevated Aβ (%)	263 (87)
Median MMSE (IQR)	29 (2)
AD dementia (%)	0 (0)

Supplementary Table 21. Study Participant Characteristics (A4/LEARN Tau subset).

Model	Beta	95% CI	t-value	p-value	FDR	ΔR^2
**All**	**0.021**	**8.4×10^{-3} to 0.033**	**3.28**	**1.2×10^{-3}**	**6.9×10^{-3}**	**0.030**
Ex	-3.2×10^{-3}	-0.016 to 9.4×10^{-3}	-0.50	0.62	0.72	NA
In	1.1×10^{-3}	-0.012 to 0.014	0.16	0.87	0.90	NA
Ast	0.014	1.1×10^{-3} to 0.027	2.14	0.033	0.067	NA
**Mic**	**0.021**	**8.2×10^{-3} to 0.033**	**3.26**	**1.2×10^{-3}**	**6.9×10^{-3}**	**0.030**
Oli	9.0×10^{-3}	-3.9×10^{-3} to 0.022	1.37	0.17	0.24	NA
Opc	3.9×10^{-3}	-8.7×10^{-3} to 0.016	0.61	0.54	0.66	NA
APOE ε4	0.032	0.011 to 0.054	3.02	2.8×10^{-3}	NA	0.025
APOE ε2	-0.045	-0.087 to -3.8×10^{-3}	-2.15	0.033	NA	0.011

Supplementary Table 22. Association between cell-type-specific ADPRS and tau in A4 (n=302). Beta (effect size) corresponds to units changed in flortaucipir PET SUVR (temporal lobe composite) per 1 s.d. increase in ADPRS. ADPRS models (linear regression) were adjusted for *APOE* ε4, *APOE* ε2, age, sex, and the first three genotype principal components. For the cell-type-specific ADPRS that showed a significant association with the trait (FDR<0.025), variance explained by the PRS was captured by comparing adjusted R^2 between the linear models with and without the given PRS term (ΔR^2). For comparison, the statistics for *APOE* ε4 and ε2 from the same model as All-ADPRS (with the same covariates) were shown in the bottom two lines of the table. All p-values are two-sided. To account for multiple comparisons, false discovery rate (FDR) correction was applied across all main tests in A4, and statistically significant results (FDR<0.025) were indicated in bold. (Also see **Fig. 3**).

Model	Beta	95% CI	t-value	p-value
Ex	0.048	-0.032 to 0.13	1.18	0.24
Ast	0.013	-0.067 to 0.093	0.31	0.76
Mic	0.16	0.081 to 0.24	3.93	1.0×10^{-4}
Oli	0.057	-0.027 to 0.14	1.33	0.18

Supplementary Table 23. Association between cell-type-specific ADPRS and tau in ROSMAP CU subset (n=454). Beta (effect size) corresponds to units changed in Aβ per 1 s.d. increase in ADPRS. ADPRS models were adjusted for *APOE* ε4, *APOE* ε2, age at death, sex, genotyping platform, and the first three genotype principal components. All p-values are two-sided and not adjusted for multiple comparisons.

	A4/LEARN MRI subset (n=1266)
Mean Age, years (SD)	71.5 (4.7)
Female (%)	753 (59)
Mean Education, years (SD)	16.7 (2.6)
APOE ε4 carrier (%)	609 (48)
Mean Florbetapir, cortical SUVR (SD)	1.22 (0.22)
Mean HV, mm^3 (SD)	3774 (417)
Elevated Aβ (%)[a]	849 (67)
Median MMSE (IQR)	29 (2)
AD dementia (%)	0 (0)

Supplementary Table 24. Study Participant Characteristics (A4/LEARN structural MRI subset). Abbreviations: APOE, apolipoprotein E; HV, hippocampal volume; IQR, interquartile range; MMSE, Mini-Mental State Examination; SD, standard deviation; SUVR, standardized uptake value ratio (whole cerebellar reference). [a]n=1265 with data.

Model	Beta	95% CI	t-value	p-value	FDR	ΔR^2
**All**	**-33**	**-52 to -14**	**-3.48**	**5.3×10^{-4}**	**4.9×10^{-3}**	**5.9×10^{-3}**
Ex	-14	-34 to 4.8	-1.47	0.14	0.21	NA
In	3.1	-16 to 22	0.32	0.75	0.84	NA
Ast	-15	-34 to 4.3	-1.52	0.13	0.21	NA
Mic	-15	-34 to 4.2	-1.53	0.13	0.21	NA
Oli	-23	-42 to -4.1	-2.39	0.017	0.041	NA
Opc	-14	-33 to 4.6	-1.49	0.14	0.21	NA
APOE ε4	-54	-86 to -22	-3.32	9.2×10^{-4}	NA	5.4×10^{-3}
APOE ε2	-24	-84 to 35	-0.81	0.42	NA	NA

Supplementary Table 25. Association between cell-type-specific ADPRS and hippocampal volume (HV) in A4 (n=1,266). Beta (effect size) corresponds to units changed in HV (mm^3) per 1 s.d. increase in ADPRS. ADPRS models (linear regression) were adjusted for *APOE* ε4, *APOE* ε2, age, sex, intracranial volume (ICV), and the first three genotype principal components. For the cell-type-specific ADPRS that showed a significant association with the trait (FDR<0.025), variance explained by the PRS was captured by comparing adjusted R^2 between the linear models with and without the given PRS term (ΔR^2). For comparison, the statistics for *APOE* ε4 and ε2 from the same model as All-ADPRS (with the same covariates) were shown in the bottom two lines of the table. All p-values are two-sided. To account for multiple comparisons, false discovery rate (FDR) correction was applied across all main tests in A4, and statistically significant results (FDR<0.025) were indicated in bold. (Also see **Fig. 3**).

Model	Beta	95% CI	t-value	p-value	FDR	ΔR^2
**All**	**-0.13**	**-0.21 to -0.045**	**-3.02**	**2.5×10^{-3}**	**0.010**	**2.3×10^{-3}**
Ex	-0.027	-0.11 to 0.056	-0.64	0.52	0.66	NA
In	-0.095	-0.18 to -0.013	-2.26	0.024	0.051	NA
**Ast**	**-0.12**	**-0.20 to -0.037**	**-2.83**	**4.6×10^{-3}**	**0.014**	**2.0×10^{-3}**
Mic	-0.088	-0.17 to -5.6×10^{-3}	-2.10	0.036	0.068	NA
Oli	-2.2×10^{-3}	-0.084 to 0.080	-0.053	0.96	0.96	NA
Opc	-0.053	-0.14 to 0.030	-1.26	0.21	0.28	NA
APOE ε4	-0.26	-0.42 to -0.11	-3.42	6.4×10^{-4}	NA	3.0×10^{-3}
APOE ε2	-0.013	-0.25 to 0.22	-0.11	0.91	NA	NA

Supplementary Table 26. Association between cell-type-specific ADPRS and Preclinical Alzheimer Cognitive Composite (PACC) in A4 (n=2,918). Beta (effect size) corresponds to units changed in PACC per 1 s.d. increase in ADPRS. ADPRS models (linear regression) were adjusted for *APOE* ε4, *APOE* ε2, age, sex, years of education, and the first three genotype principal components. For the cell-type-specific ADPRS that showed a significant association with the trait (FDR<0.025), variance explained by the PRS was captured by comparing adjusted R^2 between the linear models with and without the given PRS term (ΔR^2). For comparison, the statistics for *APOE* ε4 and ε2 from the same model as All-ADPRS (with the same covariates) were shown in the bottom two lines of the table. All p-values are two-sided. To account for multiple comparisons, false discovery rate (FDR) correction was applied across all main tests in A4, and statistically significant results (FDR<0.025) were indicated in bold. (Also see **Fig. 3**).

Phenotype	Cell Type (Genomic Margin)	Beta	95% CI	t-value	p-value
Aβ	Ex (10 kb)	8.5×10^{-3}	2.1×10^{-3} to 0.015	2.61	9.1×10^{-3}
Aβ	Ex (100 kb)	8.6×10^{-3}	2.2×10^{-3} to 0.015	2.62	8.9×10^{-3}
Aβ	Ast (10 kb)	8.0×10^{-3}	1.6×10^{-3} to 0.014	2.45	0.014
Aβ	Ast (100 kb)	9.6×10^{-3}	3.1×10^{-3} to 0.016	2.92	3.5×10^{-3}
Aβ	Mic (10 kb)	0.015	8.2×10^{-3} to 0.021	4.48	7.8×10^{-6}
Aβ	Mic (100 kb)	0.017	0.011 to 0.024	5.35	9.3×10^{-8}
Aβ	Oli (10 kb)	9.0×10^{-3}	2.6×10^{-3} to 0.015	2.75	5.9×10^{-3}
Aβ	Oli (100 kb)	9.9×10^{-3}	3.5×10^{-3} to 0.016	3.03	2.5×10^{-3}
Tau	Mic (10 kb)	0.016	3.3×10^{-3} to 0.028	2.50	0.013
Tau	Mic (100 kb)	0.020	7.8×10^{-3} to 0.033	3.19	1.6×10^{-3}
PACC	Ast (10 kb)	-0.11	-0.20 to -0.031	-2.69	7.2×10^{-3}
PACC	Ast (100 kb)	-0.12	-0.20 to -0.033	-2.76	5.9×10^{-3}

Supplementary Table 27. Association between cell-type-specific ADPRS using different genomic margins and AD endophenotypes in A4. For the significant findings using cell-type-specific ADPRS using ± 30kb margins (FDR<0.025 in **Fig. 2**), we performed sensitivity analyses using cell-type-specific ADPRS using different genomic margins (genes ± 10kb or ± 100kb). All models (linear regression) were adjusted for *APOE* ε4, *APOE* ε2, age, sex, years of education (for PACC outcome), and the first three genotype principal components. All p-values are two-sided and not adjusted for multiple comparisons.

Phenotype	Cell Type	Beta	95% CI	t-value	p-value
Aβ	Ex	8.6×10^{-3}	2.2×10^{-3} to 0.015	2.63	8.6×10^{-3}
Aβ	Ast	9.7×10^{-3}	3.3×10^{-3} to 0.016	2.96	3.1×10^{-3}
Aβ	Mic	0.017	0.011 to 0.024	5.31	1.2×10^{-7}
Aβ	Oli	9.9×10^{-3}	3.5×10^{-3} to 0.016	3.02	2.6×10^{-3}
Tau	Mic	0.023	9.9×10^{-3} to 0.036	3.49	5.6×10^{-4}
PACC	Ast	-0.12	-0.20 to -0.036	-2.83	4.7×10^{-3}

Supplementary Table 28. Association between cell-type-specific ADPRS and AD endophenotypes in A4, using 10 genotype PCs. For the significant findings from the A4 main analyses (FDR<0.025 in **Fig. 4**), we performed sensitivity analyses adjusting for 10 genotype PCs (instead of 3 PCs adjusted in the main analyses). All models (linear regression) were adjusted for *APOE* ε4, *APOE* ε2, age, sex, years of education (for PACC outcome), and the first three genotype principal components. All p-values are two-sided and not adjusted for multiple comparisons.

Phenotype	Cell Type	Beta	95% CI	t-value	p-value	ΔR^2 (PRSet)	ΔR^2 (PRSCS)
Aβ	Ex	2.1×10^{-3}	-4.4×10^{-3} to 8.5×10^{-3}	0.63	0.53	NA	1.6×10^{-3}
Aβ	Ast	2.9×10^{-3}	-3.5×10^{-3} to 9.4×10^{-3}	0.89	0.37	NA	2.1×10^{-3}
Aβ	Mic	9.6×10^{-3}	3.2×10^{-3} to 0.016	2.94	3.3×10^{-3}	2.2×10^{-3}	7.7×10^{-3}
Aβ	Oli	5.2×10^{-3}	-1.2×10^{-3} to 0.12	1.60	0.11	NA	2.3×10^{-3}
Tau	Mic	0.011	-1.8×10^{-3} to 0.023	1.68	0.095	NA	0.030
PACC	Ast	-0.065	-0.15 to 0.018	-1.54	0.12	NA	2.0×10^{-3}

Supplementary Table 29. Association between PRSet-derived ADPRS and AD endophenotypes in A4. For the significant findings from the A4 main analyses (FDR<0.025 in **Fig. 4**), we benchmarked our approach against PRSet. For the PRSet-derived ADPRS that showed a nominal association with the trait (uncorrected $p<0.05$), variance explained by the PRS was captured by comparing adjusted R^2 between the linear models with and without the given PRS term (ΔR^2). ΔR^2 from the main results (using PRS-CS) were shown for comparison. (see **Fig. 4** and **Supplementary Tables 18, 22, 25, 26**). All models (linear regression) were adjusted for *APOE* ε4, *APOE* ε2, age, sex, years of education (for PACC outcome), and the first three genotype principal components. All p-values are two-sided and not adjusted for multiple comparisons. All p-values are two-sided and not adjusted for multiple comparisons.

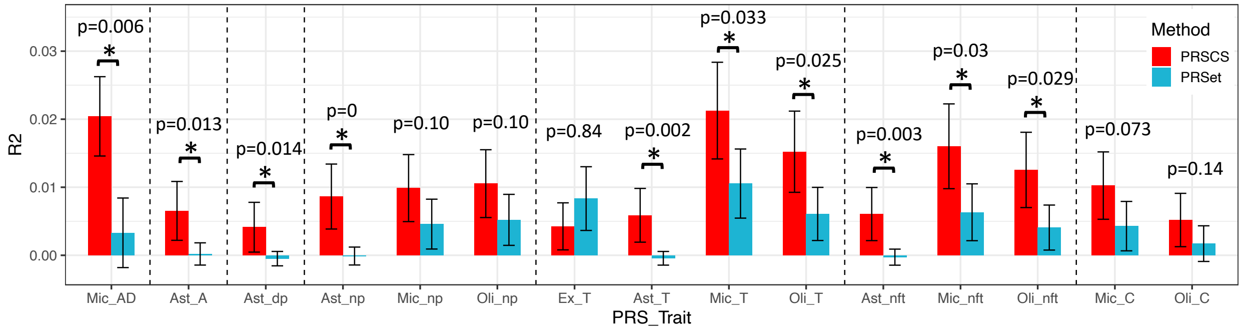

Supplementary Fig 1. ΔR^2 of significant main results (ROSMAP, PRS-CS) and PRSet-derived results. Variance explained by the PRS was captured by comparing adjusted R^2 between the linear models with and without the given PRS term (ΔR^2; Nagelkerke's R^2 was used for the AD dementia logistic regression model [first column]). The main results from this study (red bars) and the results derived with an alternative method (light blue bars) are shown. Error bars indicate bootstrap-estimated standard errors (n=1,000 bootstrap replicates). Empiric p-values, defined as the proportion of bootstrapped PRSet ΔR^2 greater than the actual PRS-CS ΔR^2, were noted above each pair of bar graphs. Asterisks indicate empiric $p<0.05$. Horizontal dashed lines separate between traits. Abbreviations: AD, AD dementia; A, amyloid-β; dp, diffuse plaque; np, neuritic plaque; T, tau; nft, neurofibrillary tangle; C, cognitive decline.

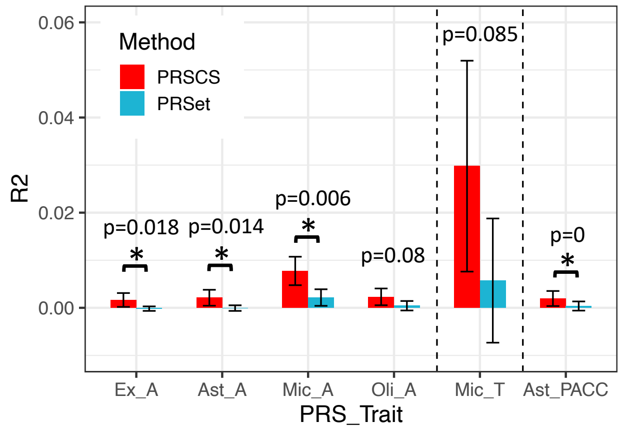

Supplementary Fig 2. ΔR^2 of significant main results (A4, PRS-CS) and PRSet-derived results. Variance explained by the PRS was captured by comparing adjusted R^2 between the linear models with and without the given PRS term (ΔR^2). The main results from this study (red bars) and the results derived with an alternative method (light blue bars) are shown. Error bars indicate bootstrap-estimated standard errors (n=1,000 bootstrap replicates). Empiric p-values, defined as the proportion of bootstrapped PRSet ΔR^2 greater than the actual PRS-CS ΔR^2, were noted above each pair of bar graphs. Asterisks indicate empiric $p<0.05$. Horizontal dashed lines separate between traits. Abbreviations: AD, AD dementia; A, amyloid-β; T, tau; HV, hippocampal volume; PACC, preclinical Alzheimer cognitive composite.
