# Supplementary material for: Cell-type-specific Alzheimer’s disease polygenic risk scores are associated with distinct disease processes in Alzheimer’s disease
Source: Nat Commun. 2023 Nov 30;14:7659. doi: 10.1038/s41467-023-43132-2 (PMC10689816; doi:10.1038/s41467-023-43132-2)
Supplement: Supplementary file 3 — Reporting Summary [file 41467_2023_43132_MOESM3_ESM.pdf]

nature portfolio

Corresponding author(s): Hyun-Sik Yang

Last updated by author(s): Oct 25, 2023

Reporting Summary

Nature Portfolio wishes to improve the reproducibility of the work that we publish. This form provides structure for consistency and transparency in reporting. For further information on Nature Portfolio policies, see our Editorial Policies and the Editorial Policy Checklist.

Statistics

For all statistical analyses, confirm that the following items are present in the figure legend, table legend, main text, or Methods section.

n/a	Confirmed	
☐	☒	The exact sample size (*n*) for each experimental group/condition, given as a discrete number and unit of measurement
☐	☒	A statement on whether measurements were taken from distinct samples or whether the same sample was measured repeatedly
☐	☒	The statistical test(s) used AND whether they are one- or two-sided<br>*Only common tests should be described solely by name; describe more complex techniques in the Methods section.*
☐	☒	A description of all covariates tested
☐	☒	A description of any assumptions or corrections, such as tests of normality and adjustment for multiple comparisons
☐	☒	A full description of the statistical parameters including central tendency (e.g. means) or other basic estimates (e.g. regression coefficient) AND variation (e.g. standard deviation) or associated estimates of uncertainty (e.g. confidence intervals)
☐	☒	For null hypothesis testing, the test statistic (e.g. *F*, *t*, *r*) with confidence intervals, effect sizes, degrees of freedom and *P* value noted<br>*Give P values as exact values whenever suitable.*
☒	☐	For Bayesian analysis, information on the choice of priors and Markov chain Monte Carlo settings
☒	☐	For hierarchical and complex designs, identification of the appropriate level for tests and full reporting of outcomes
☐	☒	Estimates of effect sizes (e.g. Cohen's *d*, Pearson's *r*), indicating how they were calculated

Our web collection on statistics for biologists contains articles on many of the points above.

Software and code

Policy information about availability of computer code

Data collection	No primary data collection was performed in this study, and no data collection software was used.
Data analysis	All statistical analyses were done with R version 4.2 (https://cran. r-project.org/). We used R packages boot, fmsb, ggplot2, lavaan, mediation, land UpSetR (detailed in the Methods section). We also used PRS-CS and PLINK (version 1.9).

For manuscripts utilizing custom algorithms or software that are central to the research but not yet described in published literature, software must be made available to editors and reviewers. We strongly encourage code deposition in a community repository (e.g. GitHub). See the Nature Portfolio guidelines for submitting code & software for further information.

Data

Policy information about availability of data

All manuscripts must include a data availability statement. This statement should provide the following information, where applicable:

- Accession codes, unique identifiers, or web links for publicly available datasets
- A description of any restrictions on data availability
- For clinical datasets or third party data, please ensure that the statement adheres to our policy

Data Availability: ROSMAP phenotype data (demographic, neuropathology, diagnoses, and cognitive testing data) can be requested at the RADC Resource Sharing Hub at https://www.radc.rush.edu. ROSMAP genotype data can be requested at the AD Knowledge Portal under accession code syn23446022 (https://www.synapse.org/#!Synapse:syn23446022; see https://adknowledgeportal.synapse.org/Data%20Access for data access instructions). The A4/LEARN screening (pre-randomization) data (demographic, neuroimaging, cognitive testing, and genetic data) can be requested at https://ida.loni.usc.edu/. All of the primary data used in

this study are individual-level human data that require the investigators to sign a data use agreement (ROSMAP phenotype and all A4 data) or a data use certificate (ROSMAP genotype data) to ensure human subject protection; data access instructions can be found in the above URLs. We made the PRS-CS posterior effect sizes of AD GWAS summary statistics and cell-type-specific gene tracks (genomic ranges; each track defines the list of SNPs used for each PRS) available at the AD knowledge portal under accession code syn52750861 as open data (DOI: https://doi.org/10.7303/syn52750861). Source data are provided with this paper.

Research involving human participants, their data, or biological material

Policy information about studies with human participants or human data. See also policy information about sex, gender (identity/presentation), and sexual orientation and race, ethnicity and racism.

Reporting on sex and gender	We have considered biological sex in our study design. Reported sex was confirmed with genotype-inferred sex. (Gender was not separately assessed.) We have adjusted sex in all our analyses, and we have also tested sex interaction to examine for sex differences in reported effects. Please note that sharing of individual-level data from the parent studies require data use agreement or data use certificate, as noted in Data Access statement. (We have not observed statistically significant sex differences per statistical interaction analyses, and we have reported this in the manuscript.)
Reporting on race, ethnicity, or other socially relevant groupings	Population outliers, including all participants of non-European descent, were not included in this study to avoid confounding by population structure.
Population characteristics	We provide demographic characteristics of our study participants in Table 1.
Recruitment	ROSMAP: The Religious Orders Study (ROS) and the Rush Memory and Aging Project (MAP) were approved by an Institutional Review Board (IRB) of Rush University Medical Center. The ROS started in 1994 and is enrolling Catholic priests, brothers, and nuns across religious communities in the United States. The MAP started in 1997 and is enrolling diverse participants from northeastern Illinois. Each participant signed an informed consent, Anatomic Gift Act, and Repository Consent allowing their data to be repurposed. Both studies enrolled older participants who did not have known dementia at enrollment and agreed to organ donation after death (overall autopsy rate > 85%).<br>A4: The A4 study protocol was approved by IRBs at each participating site, and all participants signed informed consent before the study procedures. The A4 study is a secondary prevention trial that enrolled CU older adults (between age 65 and 85) with evidence of cortical Aβ accumulation on PET imaging from 67 sites in the United States, Australia, Canada, and Japan. Inclusion criteria to select CU older adults included Clinical Dementia Rating global score of 0, Mini-Mental State Examination (MMSE) score of 25 to 30, and Logical Memory Delayed Recall (LMDR) score of 6 to 18.
Ethics oversight	ROSMAP: Institutional Review Board (IRB) of Rush University Medical Center<br>A4: IRBs at each participating site (67 sites worldwide)

Note that full information on the approval of the study protocol must also be provided in the manuscript.

Field-specific reporting

Please select the one below that is the best fit for your research. If you are not sure, read the appropriate sections before making your selection.

- [x] Life sciences
- [ ] Behavioural & social sciences
- [ ] Ecological, evolutionary & environmental sciences

For a reference copy of the document with all sections, see nature.com/documents/nr-reporting-summary-flat.pdf

Life sciences study design

All studies must disclose on these points even when the disclosure is negative.

Sample size	We have included all parent study (ROSMAP, A4) participants with non-missing values to maximize sample size; no statistical method was used to predetermine sample size. ROSMAP n=1457, A4 n=2921.
Data exclusions	Participants with missing values were excluded from each analysis, and we indicated the number of participants for each analysis. The analyses was limited to individuals of European descent as the base GWAS summary statistics was derived from the GWAS on individuals of European descent and does not extrapolate to non-European ancestries.
Replication	(1) We have analyzed different measures of similar endophenotype in two independent datasets (e.g., post-mortem AD pathology in ROSMAP vs. neuroimaging biomarkers of AD pathology in A4), and observed that key associations are consistently observed (e.g., microglial ADPRS - tau; astrocytic ADPRS - amyloid-beta). (2) We performed our analyses using varying parameters to derive cell-type-specific PRS (size of genomic margin, number of genotype principal components adjusted in the model) to rule out parameter-driven results. (3) We have accounted for multiple comparisons using false discovery rate across each study.
Randomization	This study only uses observational data (ROSMAP and A4 pre-randomization data), and thus randomization was not applicable.
Blinding	This study only uses observational data (ROSMAP and A4 pre-randomization data), and thus blinding was not applicable.

Reporting for specific materials, systems and methods

We require information from authors about some types of materials, experimental systems and methods used in many studies. Here, indicate whether each material, system or method listed is relevant to your study. If you are not sure if a list item applies to your research, read the appropriate section before selecting a response.

Materials & experimental systems

n/a	Involved in the study
☒	☐ Antibodies
☒	☐ Eukaryotic cell lines
☒	☐ Palaeontology and archaeology
☒	☐ Animals and other organisms
☒	☐ Clinical data
☒	☐ Dual use research of concern
☒	☐ Plants

Methods

n/a	Involved in the study
☒	☐ ChIP-seq
☒	☐ Flow cytometry
☒	☐ MRI-based neuroimaging

Plants

Seed stocks	*Report on the source of all seed stocks or other plant material used. If applicable, state the seed stock centre and catalogue number. If plant specimens were collected from the field, describe the collection location, date and sampling procedures.*
Novel plant genotypes	*Describe the methods by which all novel plant genotypes were produced. This includes those generated by transgenic approaches, gene editing, chemical/radiation-based mutagenesis and hybridization. For transgenic lines, describe the transformation method, the number of independent lines analyzed and the generation upon which experiments were performed. For gene-edited lines, describe the editor used, the endogenous sequence targeted for editing, the targeting guide RNA sequence (if applicable) and how the editor was applied.*
Authentication	*Describe any authentication procedures for each seed stock used or novel genotype generated. Describe any experiments used to assess the effect of a mutation and, where applicable, how potential secondary effects (e.g. second site T-DNA insertions, mosiacism, off-target gene editing) were examined.*
